# A Selective Bottleneck During Host Entry Drives the Evolution of New Legume Symbionts

**DOI:** 10.1093/molbev/msad116

**Published:** 2023-05-15

**Authors:** Ginaini Grazielli Doin de Moura, Saida Mouffok, Nil Gaudu, Anne-Claire Cazalé, Marine Milhes, Tabatha Bulach, Sophie Valière, David Roche, Jean-Baptiste Ferdy, Catherine Masson-Boivin, Delphine Capela, Philippe Remigi

**Affiliations:** LIPME, Université de Toulouse, INRAE, CNRS, Castanet-Tolosan, France; LIPME, Université de Toulouse, INRAE, CNRS, Castanet-Tolosan, France; LIPME, Université de Toulouse, INRAE, CNRS, Castanet-Tolosan, France; LIPME, Université de Toulouse, INRAE, CNRS, Castanet-Tolosan, France; INRAE, US1426, GeT-PlaGe, Genotoul, Castanet-Tolosan, France; INRAE, US1426, GeT-PlaGe, Genotoul, Castanet-Tolosan, France; INRAE, US1426, GeT-PlaGe, Genotoul, Castanet-Tolosan, France; Génomique Métabolique, Genoscope, Institut François Jacob, CEA, CNRS, Univ Evry, Université Paris-Saclay, Evry, France; Evolution et Diversité Biologique, UMR5174, CNRS-Université Paul Sabatier, Toulouse, France; LIPME, Université de Toulouse, INRAE, CNRS, Castanet-Tolosan, France; LIPME, Université de Toulouse, INRAE, CNRS, Castanet-Tolosan, France; LIPME, Université de Toulouse, INRAE, CNRS, Castanet-Tolosan, France

**Keywords:** rhizobium, experimental evolution, symbiosis, population bottleneck, adaptation

## Abstract

During the emergence of new host–microbe symbioses, microbial fitness results from the ability to complete the different steps of symbiotic life cycles, where each step imposes specific selective pressures. However, the relative contribution of these different selective pressures to the adaptive trajectories of microbial symbionts is still poorly known. Here, we characterized the dynamics of phenotypic adaptation to a simplified symbiotic life cycle during the experimental evolution of a plant pathogenic bacterium into a legume symbiont. We observed that fast adaptation was predominantly explained by improved competitiveness for host entry, which outweighed adaptation to within-host proliferation. Whole-population sequencing of bacteria at regular time intervals along this evolution experiment revealed the continuous accumulation of new mutations (fuelled by a transient hypermutagenesis phase occurring at each cycle before host entry, a phenomenon described in previous work) and sequential sweeps of cohorts of mutations with similar temporal trajectories. The identification of adaptive mutations within the fixed mutational cohorts showed that several adaptive mutations can co-occur in the same cohort. Moreover, all adaptive mutations improved competitiveness for host entry, while only a subset of those also improved within-host proliferation. Computer simulations predict that this effect emerges from the presence of a strong selective bottleneck at host entry occurring before within-host proliferation and just after the hypermutagenesis phase in the rhizosphere. Together, these results show how selective bottlenecks can alter the relative influence of selective pressures acting during bacterial adaptation to multistep infection processes.

## Introduction

Many bacterial lineages have evolved the capacity to establish symbiotic associations, either beneficial, neutral, or parasitic, with eukaryotic hosts. These interactions form a dynamic continuum along which bacteria can move (Drew et al. 2021). Lifestyle changes may arise due to ecological (resource availability, host environment changes, or host shifts) or genomic (mutations and acquisition of new genetic material) modifications. These new interactions are often initially suboptimal for the bacterial partner (Nandasena et al. 2007; Longdon et al. 2015; Bonneaud and Longdon 2020), which may then adapt to the selective pressures associated with its new life cycle. For instance, horizontally transmitted microbes alternate between at least two different habitats, the host and the environment, where they will face a variety of constraints and selective pressures (Robinson et al. 2019; Obeng et al. 2021). Indeed, to complete biphasic life cycles, bacteria need not only to enter and exit their hosts but also to replicate and persist within each habitat, which entails adapting to abiotic stressors, host immunity, competitors, and specific nutrient sources. However, the relative influence of the different selective pressures on the dynamics and the trajectory of bacterial adaptation to a new interaction is poorly known.

Rhizobia are examples of facultative host-associated bacteria that can either live freely in soil or in symbiotic mutualistic associations with legume plants (Wheatley et al. 2020). In most legumes, rhizobia penetrate the root tissue through the formation of so-called infection threads (ITs). Bacteria then divide within ITs, while at the same time a nodule starts to develop at the basis of the infected root hair. Bacteria are then released from ITs inside the cells of the developing nodule and differentiate into nitrogen fixing bacteroids (Gage 2004; Coba de la Peña et al. 2017). When legume plants are coinoculated with two bacterial genotypes, bacterial populations within nodules are mostly clonal (Gage 2002; Daubech et al. 2017), which suggests that each nodule is founded by one single bacterial cell. Since only tens to hundreds of nodules can be formed on the same plant in nature, a strong population bottleneck occurs between large rhizospheric bacterial populations and bacteria entering root tissues. After several months, in nature, nodule senescence leads to the release of a part of bacterial nodule population in the surrounding soil. Up to hundreds of millions of viable bacteria can be released from each nodule (Downie 2014). To fulfill their life cycle, rhizobia rely on the activity of numerous bacterial genes, allowing signal exchanges with the host plant, proliferation, and metabolic exchanges within nodule cells, while maintaining free-living proficiency (Wheatley et al. 2020). Somewhat surprisingly given their complex and specialized lifestyle, rhizobia evolved several times independently. Horizontal transfer of key symbiotic genes is a necessary, though often not sufficient, condition for a new rhizobium to emerge (Hirsch et al. 1984; Abe et al. 1998; Nakatsukasa et al. 2008; Marchetti et al. 2010). Additional steps of adaptation of the recipient bacterium, occurring during evolution under plant selection, are believed to be needed to actualize the symbiotic potential of emerging rhizobia when the recipient genotype is not already compatible with legume symbiosis (Masson-Boivin et al. 2009).

In a previous study, we used experimental evolution to convert the plant pathogen *Ralstonia solanacearum* into an intracellular legume symbiont (Doin de Moura et al. 2020). In this experiment, we transferred a symbiotic plasmid (pRalta) from the rhizobium *Cupriavidus taiwanensis* LMG19424, the *Mimosa pudica* symbiont, into the GMI1000 strain of *R. solanacearum*. We first obtained three nodulating variants, two infecting nodules intracellularly (CBM212 and CBM349) and one infecting nodules extracellularly (CBM356) (Marchetti et al. 2010), which were used as ancestors to evolve 18 parallel bacterial lineages through 16 serial cycles of nodulation on *M. pudica* plants (Marchetti et al. 2017). Some highly adaptive mutations involved in the acquisition of nodulation and intracellular infection of nodules were previously identified (Marchetti et al. 2010; Guan et al. 2013; Capela et al. 2017; Tang et al. 2020). However, these previous works only gave us a partial and nondynamic view of the adaptive process at both phenotypic and molecular levels. Here, we analyzed the dynamics of phenotypic and molecular evolution in five lineages evolved for 35 cycles of nodulation. We used whole-population genome sequencing and examined the selective and genetic bases of adaptation. Adaptation proceeded rapidly during the first cycles of evolution and was underpinned by the fixation of successive cohorts of mutations within populations. We then identified adaptive mutations in two lineages and evaluated their effect on three symbiotic stages of the rhizobial life cycle, rhizosphere colonization, nodule formation, and proliferation inside nodules. Our experimental data indicated that selection for nodulation competitiveness outweighs selection for multiplication within host. Computer simulations further showed that the selective bottleneck at host entry and the chronology of symbiotic events are critical drivers of this evolutionary pattern.

## Results

### Fast Adaptation of New Legume Symbionts during the First Cycles of Evolution

**Fig. 1.**
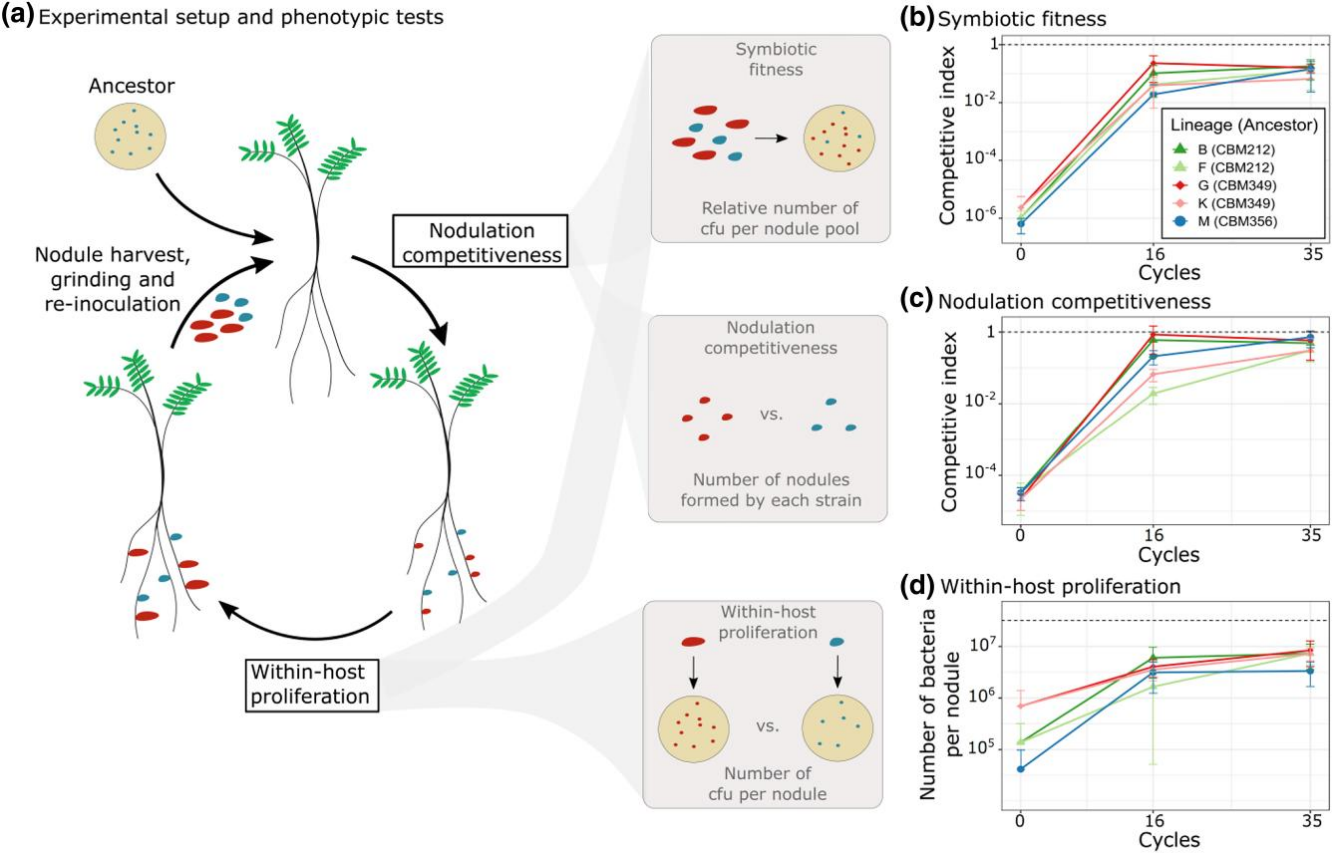
Evolution of the symbiotic properties of *Ralstonia* clones along evolution cycles. (*a*) Left: overview of the evolution cycles showing the symbiotic steps determining bacterial fitness (nodulation competitiveness and within-host proliferation) and human intervention (nodule harvest, grinding, and reinoculation). Cycle lengths were 21 days for lines B, G, and M and 42 days for lines F and K until cycle 16 and then 21 days until cycle 35. Nodule colors reflect bacterial genotypes: Blue represents the ancestor, and red represents a mutant with increased fitness. Right: schematic representation of the main phenotypic measurements performed in this work, where the symbiotic phenotypes of one evolved strain can be compared to those of a reference strain (e.g., the reference symbiont *C. taiwanensis*). CIs for symbiotic fitness were calculated as the relative frequencies of evolved clones and the reference strain in nodule bacterial populations normalized by the relative frequency of the two strains in the inoculum. CIs for nodulation competitiveness were calculated as the ratio of nodules formed by each strain normalized by the frequency of each strain in the inoculum. Within-host proliferation is measured in independent single inoculations of each strain. (*b*–*d*) Relative symbiotic fitness (*b*), nodulation competitiveness (*c*), and within-host proliferation (*d*) of nodulating ancestors (cycle 0) and evolved clones isolated from cycles 16 and 35 were compared with *C. taiwanensis*. Values correspond to means ± standard deviations. Dotted lines correspond to CIs equal to 1 (*b* and *c*) or to the number of bacteria per nodule recovered for the reference strain *C. taiwanensis* (*d*). Data were obtained from at least three independent experiments. For each experiment, nodules were harvested from 10 plants (*b*), 20 plants (*c*), and 6 plants (*d*). The sample size (*n*) is equal to *n* = 3 (*b* and *c*) or comprised between *n* = 15 and 18 (*d*). cfu, colony-forming units. The data underlying panels *b*–*d* can be found in supplementary table S12, Supplementary Material online.

To investigate the genetic and evolutionary conditions that promote the evolution of new rhizobia, we previously experimentally evolved the plant pathogen *R. solanacearum* GMI1000 into intracellular *M. pudica* symbionts through 16 serial cycles of nodulation on *M. pudica* plants (Marchetti et al. 2017). Here, we continued the evolution of five lineages (referred to as lineages B, F, G, K, and M) until cycle 35 (fig. 1*a* and supplementary fig. S1 and table S1, Supplementary Material online). After 35 cycles, no nitrogen fixation sustaining plant growth was observed (supplementary fig. S2, Supplementary Material online). Therefore, in this manuscript, we focused exclusively on the analysis of changes in bacterial symbiotic fitness (estimated for each strain as the frequency of bacteria present in nodule pools collected from ten plants relative to a competing reference strain and normalized by the frequency of each strain in the inoculum). To analyze the dynamics of fitness changes over time, we compared the relative fitness of the nodulating ancestors and evolved clones from cycles 16 and 35 by replaying one nodulation cycle in competition with the natural symbiont *C. taiwanensis.* Fitness trajectories were similar in the five lineages. Although the three nodulating ancestors were on average 10^6^ times less fit than *C. taiwanensis*, fitness improved very quickly during the first 16 cycles and then slower until cycle 35 (fig. 1*b*). On average, evolved clones isolated at cycle 16 and cycle 35 were 47 and 19 times less fit than *C. taiwanensis*, respectively (fig. 1*b*).

Because bacterial symbiotic fitness in our system depends on both the capacity of strains to enter the host and induce nodule formation (nodulation competitiveness) and to multiply within these nodules (within-host proliferation), we analyzed how each of these two fitness components changed during the experiment (fig. 1*c* and *d*). In the five lineages, evolutionary trajectories of nodulation competitiveness resembled that of fitness with fast improvement during the first 16 cycles and a slowdown during the last cycles. On average, nodulating ancestors were 5 × 10^4^ times less competitive than *C. taiwanensis*, while cycle 16 and cycle 35 evolved clones were only 17 and 4 times less competitive than *C. taiwanensis*, respectively (fig. 1*c*). Moreover, evolved clones B16, B35, G16, G35, and M35 were not statistically different from *C. taiwanensis* in terms of nodulation competitiveness. Within-host proliferation also improved mostly during the first 16 cycles in all lineages. The rate of improvement then slowed down in two lineages and continued to increase significantly in the three others. Consistent with previously published data, the three nodulating ancestors display different capacities to infect nodules (Marchetti et al. 2010, 2017), the extracellularly infective ancestor CBM356 being the less infective (750 times less than *C. taiwanensis*), and the intracellularly infective CBM349 being the most infective (45 times less than *C. taiwanensis*). On average, cycle 16 and cycle 35 evolved clones were eight and five times less infective than *C. taiwanensis* (fig. 1*d*).

Altogether, the symbiotic properties of evolved clones improved rapidly during the first cycles of the evolution experiment, and then adaptation slowed down. Both symbiotic traits, nodulation competitiveness (i.e., host entry) and within-host proliferation, improved greatly. However, gains in nodulation competitiveness (average factor of 11,500 between ancestors and cycle 35 clones) were ∼150 times higher than gains in proliferation (average factor of 74 between ancestors and cycle 35 clones). Moreover, although the difference in nodulation competitiveness between *C. taiwanensis* and the nodulating ancestors was much greater (>10^4^ fold) than the difference in proliferation (<10^3^ fold), nodulation competitiveness of evolved clones reached the level of *C. taiwanensis* in three lineages (B, G, and M) while within-host proliferation remained lower than *C. taiwanensis* in all of them after 35 cycles.

### The Dynamics of Molecular Evolution Is Characterized by Multiple Selective Sweeps of Large Mutational Cohorts

The rapid adaptation in this experiment was unexpected given that strong population bottlenecks, such as the one occurring at the nodulation step, limit effective population size at each cycle (see supplementary table S1, Supplementary Material online for inoculum population size and the numbers of nodules harvested at each cycle) and are generally known to limit the rate of adaptive evolution (Moxon and Kussell 2017). To describe the dynamics of evolution at the molecular level, we performed whole-population sequencing of evolved lineages using the Illumina sequencing technology. Populations of the five lineages were sequenced every other cycle until cycle 35 with a minimum sequencing coverage of 100× and a median of 347× (supplementary table S2, Supplementary Material online). We detected a very large number of mutations in all sequenced populations. In the five lineages, a total of 4,114 mutations were detected above a frequency of 5%, a threshold below which we considered that the detected mutations are potentially errors. This corresponds to 382–1,204 mutations per lineage with 23.6 new mutations per cycle on average (supplementary tables S3 and S4, Supplementary Material online). This high number of mutations is the result of a previously characterized transient hypermutagenesis phenomenon occurring in the rhizosphere of *M. pudica*, leading to an estimated mutation rate of 1.2–4 × 10^−10^ synonymous mutations per base pair and per generation (Remigi et al. 2014). This phenomenon is due to the presence of a cassette of error-prone DNA polymerases on the symbiotic plasmid pRalta, whose expression is induced under stressful conditions and is regulated by the SOS response. Hypermutation was specifically observed in the rhizosphere of *M. pudica* but not in nodules nor in bacterial cultures in rich medium. In evolved populations, mutations accumulated throughout the experiment, showing no sign of slowing down until the end of the experiment, which suggests that no antimutator mutations established in these populations as we might have expected (Lynch et al. 2016). We evaluated genetic parallelism among mutations detected above a frequency of 5% in the populations by computing G scores (Tenaillon et al. 2016), a statistics used to point out genes that could be mutated more often than expected by chance (supplementary table S5, Supplementary Material online). In spite of high mutation rates that may obscure signals of genetic convergence, we detected signatures of parallelism at the gene level among our list of mutations (observed sum of G scores of 7,540.5, compared with a mean sum of 5,401.01 after 1,000 randomized simulations, *Z* = 31.23, *P* < 10^−200^). The randomized simulations identified 171 genes with a higher number of mutations than expected by chance (Bonferroni adjusted *P* < 0.01; supplementary table S5, Supplementary Material online).

**Fig. 2.**
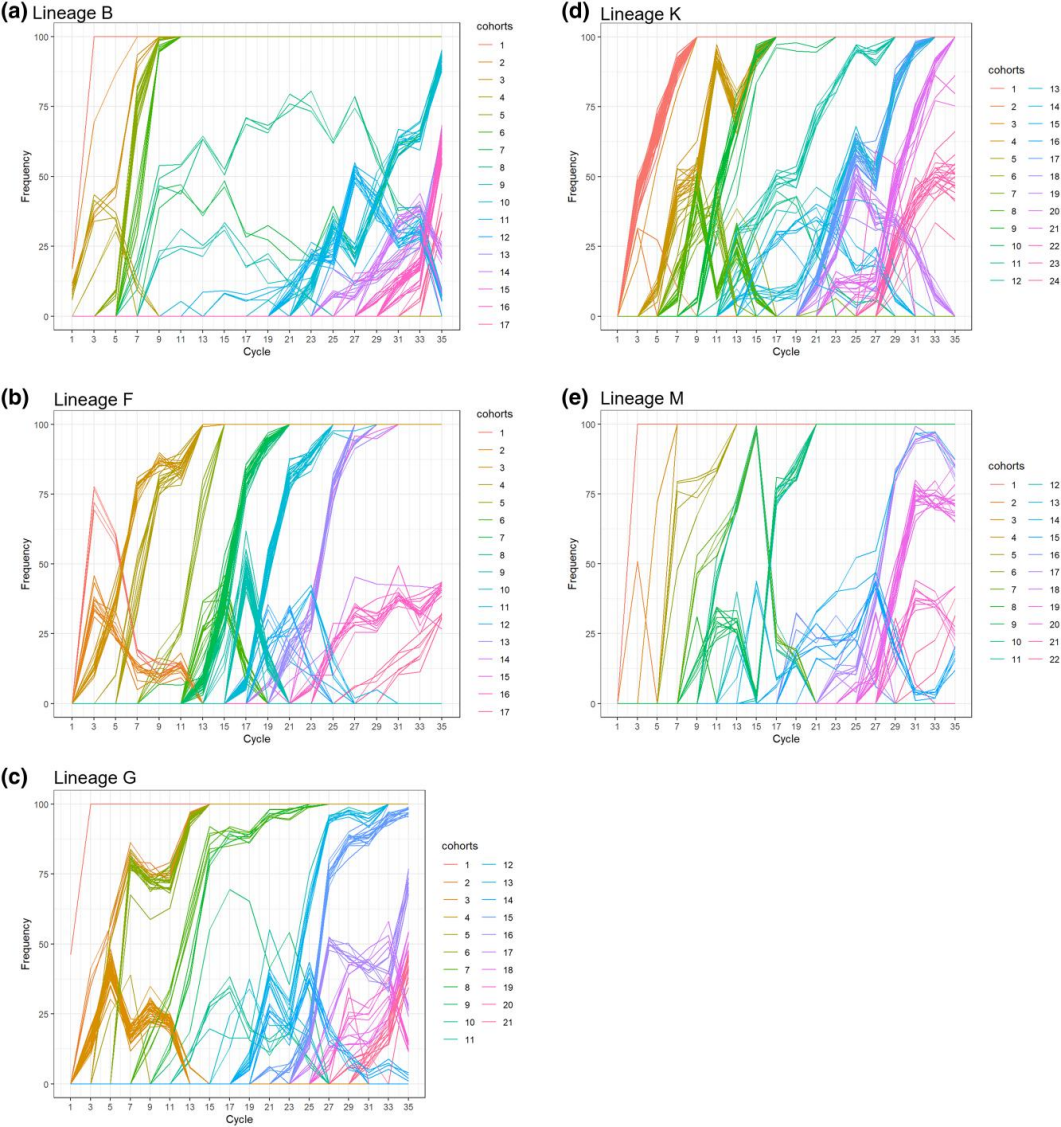
Dynamics of molecular evolution. Allele frequency trajectories of mutations that attained a frequency of 30% in at least one population of the B, F, G, K, and M lineages (*a*, *b*, *c*, *d*, and *e*). Mutations with similar trajectories were clustered in cohorts, which are represented by different colors. For simplicity, mutations travelling alone were also called cohorts. The data underlying panels *a*–*e* can be found in supplementary table S4, Supplementary Material online.

To simplify the analysis of mutational trajectories, we then focused on the 819 mutations (out of the total of 4,414 detected mutations) that rose above a frequency of 30%. These mutations did not show independent trajectories. Instead, we observed that groups of mutations arose synchronously and showed correlated temporal trajectories, enabling us to cluster them in cohorts (Lang et al. 2013; Buskirk et al. 2017) (fig. 2 and supplementary table S6, Supplementary Material online). Cohorts increased in frequency with variable speed, some being fixed within 1–3 cycles (representing *∼*25–75 bacterial generations) while others reached fixation in up to 21 cycles (representing >500 bacterial generations). Fixed cohorts can be very large (up to 30 mutations) (supplementary table S6, Supplementary Material online), which suggests hitchhiking of neutral or slightly deleterious mutations with one or several beneficial mutations acting as driver (or codrivers) of the cohort (Lang et al. 2013; Nguyen Ba et al. 2019). Among the mutations that rose above 30% frequency at some stage of the experiment, 35% later declined until extinction. This is indicative of clonal interference, that is, the co-occurrence of multiple subpopulations competing against each other within populations (McDonald 2019), which can readily be observed on Muller plots for lineages B and G (supplementary fig. S3, Supplementary Material online).

Overall, in these five lineages, the pattern of molecular evolution was characterized by a steady accumulation of mutations along the successive cycles and the formation of mutational cohorts, some of which containing a large number of mutations. Despite strong population bottlenecks at the root entry, mutation supply was not limiting as evidenced by the co-occurrence of competing adaptive mutations. Yet, multiple (and sometimes rapid) selective sweeps occurred throughout the 35 cycles in all lineages, suggesting that strong selection is acting on these populations.

### Large Fixed Cohorts Contain Multiple Adaptive Mutations

**Fig. 3.**
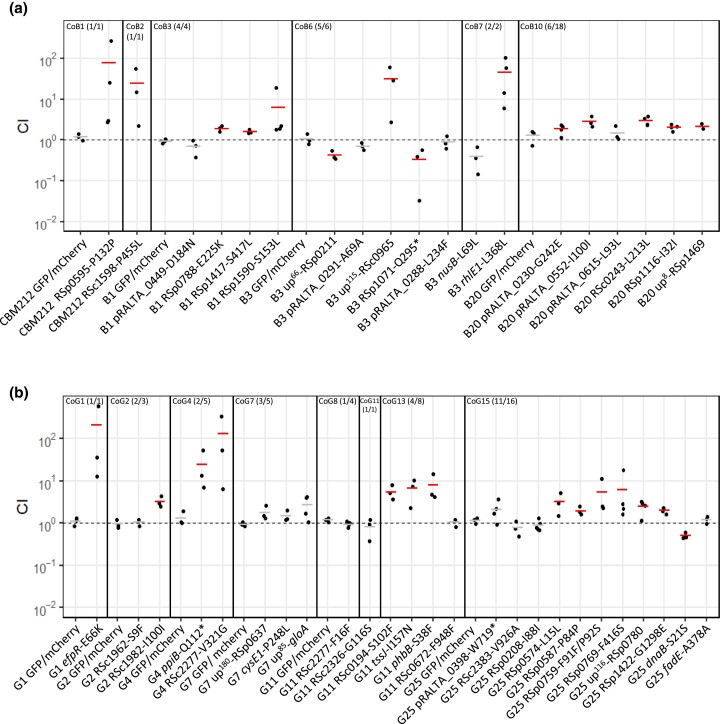
Symbiotic fitness of reconstructed mutants from lineages B and G. Relative symbiotic fitness of evolved clones carrying reconstructed mutations from fixed mutational cohorts identified in lineages B (*a*) and G (*b*). Evolved clones in which mutations were reconstructed are indicated at the bottom of the graph. Vertical lines separate the different mutational cohorts. Cohort number and the number of tested mutations from the cohort on the total number of mutations present in the cohort are indicated in brackets. CIs were calculated as the relative frequencies of the mutant strain and its isogenic parental strain in bacterial nodule populations as determined by colony fluorescence, normalized by the frequency of the each strain in the inoculum. GFP/mCherry correspond to control coinoculation experiments of strains derived from the same evolved clone labeled with different fluorophore either GFP or mCherry. Horizontal segments correspond to mean values of CI. Red segments indicate significantly beneficial (CI > 1) or deleterious (CI < 1) mutations; gray segments indicate neutral mutations (*P* < 0.05, *t*-test with Benjamini–Hochberg correction). Data were obtained from three to five independent experiments. The sample size (*n*) is comprised between *n* = 3 and 5. Each CI value was obtained from pools of 40–227 nodules harvested from ten plants. The data underlying panels *a* and *b* and *P* values can be found in supplementary table S14, Supplementary Material online.

In our system, strong nodulation bottlenecks lead to a small effective population size and to the possibility that genetic drift might be responsible for the observed allelic sweeps. To determine whether drift or adaptation is causing these sweeps, we searched for adaptive mutations in fixed (or nearly fixed, >90% frequency) cohorts from the two lineages that have the highest symbiotic fitness after 35 cycles: B and G. To do so, we introduced (by genetic engineering) individual mutations from each cohort of interest into an evolved clone carrying all (or, when not available, almost all) previously fixed cohorts. This allowed us to test the symbiotic fitness effect associated with each mutation in a relevant genetic background, taking into account possible epistatic effects arising from mutations that were previously acquired in this clone. For each mutation, competitive indexes (CIs) were calculated from the relative frequencies of mutant versus wild-type strains recovered from crushes of nodule pools harvested from ten plants and normalized by the frequency of each strain in the inoculum. In total, 44 mutations belonging to 14 cohorts from the two lineages were tested (fig. 3*a* and *b*). Twenty-five mutations significantly increased the fitness of evolved clones and were thus beneficial for symbiosis while 16 mutations were neutral and 3 were slightly deleterious for symbiosis (fig. 3*a* and *b*, table 1, and supplementary table S7, Supplementary Material online). Among the 43 genes targeted by the reconstructed mutations, eight have a higher number of mutations than expected by chance (supplementary table S5, Supplementary Material online), and adaptive mutations were identified in five of them (table 1).

**Table 1.** Symbiotic Phenotypes of Beneficial Mutations Improving Symbiotic Fitness.

Lineage	Gene ID^a^	Gene name	Mutation	Type of mutation^b^	Description	Cohort	Mean CI for in planta fitness^c^	Mean CI for proliferation in plant culture medium^d^	Mean CI for rhizosphere colonization^d^	Mean CI for nodulation competitiveness^c^	Mean gain factor in within-host proliferation^e^	Symbiotic phenotypes^f^	G score^g^	Adjusted *P* value^h^
B	RSp0595		P132P	Syn	Putative N-acyl-homoserine lactonase	CoB1	**77**	1	1	**5.2**	1.6	Nod+	2.80	1
	RSc1598		P455L	Nonsyn	Transmembrane sensor histidine kinase	CoB2	**24.2**	1	1.4	**4.7**	**6.4**	Nod+ Pro+	1.04	1
	RSp0788		E225K	Nonsyn	Carbamoyl-transferase	CoB3	**1.9**	1	1.1	**2**	1.2	Nod+	−0.09	1
	RSp1417		S417L	Nonsyn	Multidrug efflux transmembrane protein	CoB3	**1.6**	0.8	1.4	**3.9**	0.9	Nod+	12.47	**2.58E-04**
	RSp1590		S153L	Nonsyn	FAD-dependent oxireductase	CoB3	**6.3**	1	0.9	**2.4**	0.6	Nod+	4.10	1
	up^115^-RSc0965		G/A	Intergenic	Unknown function, negatively regulates *efpR* expression	CoB6	**31.1**	0.9	**2.9**	**8.2**	**2.6**	Rhizo+ Nod+ Pro+	nd	nd
	RSc0539	*rhlE1*	L368L	Syn	ATP-dependent RNA helicase protein	CoB7	**46.2**	1.1	**4**	**28.2**	**2.4**	Rhizo+ Nod+ Pro+	2.88	1
	RSc0243		L213L	Syn	LysR-type transcription regulator protein	CoB10	**3**	1.3	1.4	**3.9**	1.1	Rhizo+ Nod+	1.16	1
	RSp1116		I32I	Syn	Putative low specificity L-threonine aldolase	CoB10	**2.1**	1.2	1.2	**2.1**	0.9	Nod+	0.84	1
			L33F	Nonsyn										
	up^8^-RSp1469		C/T	Intergenic	Putative tyrosine-specific protein phosphatase	CoB10	**2.1**	0.9	1	**2**	1.0	Nod+	nd	nd
	pRALTA_0230		G242E	Nonsyn	Putative ATP-dependent DNA helicase	CoB10	**1.5**	1.5	1.5	**2.1**	0.9	Nod+	6.34	1
	pRALTA_0552		I100I	Syn	Pseudogene, putative membrane bound hydrogenase	CoB10	**2.9**	1.2	1.4	**2.3**	0.8	Nod+	nd	nd
G	RSc1097	*efpR*	E66K	Nonsyn	Transcriptional regulator	CoG1	**212.1**	1.3	**4**	**35**	**3.7**	Rhizo+ Nod+ Pro+	3.17	1
	RSc1982		I100I	Syn	DNA methyltransferase	CoG2	**3.3**	3.1	1.3	**4.0**	0.7	Nod+	1.44	1
	RSc1164	*ppiB*	Q112*	Nonsense	Peptyl-prolyl-*cis*/*trans*-isomerase	CoG4	**24.5**	1	1.0	**5.0**	1.4	Nod+	2.26	1
	RSc2277		V321G	Nonsyn	Putative transporter protein	CoG4	**133.9**	9.3	10.1	**58**	**2.5**	Nod+ Pro+	36.95	**4.68E-50**
	RSc0194		S102F	Nonsyn	Zinc-dependent alcohol dehydrogenase	CoG13	**5.5**	1.1	1.6	**3.1**	1.4	Nod+	4.67	1
	RSc1633	*phbB*	S38F	Nonsyn	Acetoacetyl-CoA reductase	CoG13	**7.9**	2	2.1	**3.3**	**1.7**	Rhizo+ Nod+ Pro+	1.62	1
	RSp0741	*tssJ*	I157N	Nonsyn	Type VI secretion system, lipoprotein TssJ	CoG13	**6.7**	1	1.1	**2.9**	1.7	Nod+	6.99	1
	RSp0574		L15L	Syn	Hypothetical protein	CoG15	**3.2**	1.1	1.3	**2.1**	**1.6**	Nod+ Pro+	4.36	1
	RSp0587		P84P	Syn	Putative signal peptide hypothetical protein	CoG15	**2**	1.1	0.8	**2.6**	1.3	Nod+	28.74	**1.04E-46**
	RSp0759		F91F	Syn	Putative type VI secretion-associated protein	CoG15	**5.4**	0.7	0.9	**2.5**	1.4	Nod+	1.00	1
			P92S	Nonsyn										
	RSp0769		F416S	Nonsyn	Putative type VI secretion-associated protein	CoG15	**6.3**	1.1	1.3	**1.6**	**1.8**	Nod+ Pro+	17.20	**3.98E-10**
	up^116^-RSp0780		C/T	Intergenic	Hypothetical protein	CoG15	**2.5**	1.1	1.1	**3**	1.1	Nod+	nd	nd
	RSp1422		G1298E	Nonsyn	Nonribosomal peptide synthase	CoG15	**2**	1.2	1.2	**2.4**	**1.6**	Nod+	18.28	**2.79E-03**

a up^x^, intergenic mutations located × nucleotides upstream the gene indicated.

b Syn, synonymous mutations; Nonsyn, nonsynonymous mutations.

c Mean values of CIs obtained from three to four independent experiments. Figures in bold are statistically different from 1 (*P* < 0.05, *t*-test with the Benjamini–Hochberg correction).

d Mean values obtained from at least 15 measurements from three to four independent experiments. Figures in bold are statistically significant (*P* < 0.05, Wilcoxon test with the Benjamini–Hochberg correction).

e Mean values obtained from at least 15 measurements from three to four independent experiments. Figures in bold are statistically different from 1 (*P* < 0.05, Wilcoxon test with Benjamini–Hochberg correction).

f Rhizo+, improvement in rhizosphere colonization; Nod+, improvement in nodulation competitiveness; Pro+, improvement in within-host proliferation.

g G scores were calculated as proposed by Tenaillon et al. (2016). nd, G scores were not determined for mutations in pseudogenes or intergenic regions.

h *P* values associated with G scores were adjusted using a Bonferroni correction to evaluate genetic parallelism.

Several interesting results emerge from this data set. First, some mutations show very strong adaptive effects, improving >100 times the fitness of bacteria, in particular during the first cycles of evolution. Second, many cohorts carry more than one adaptive mutation: five cohorts contain between two and six adaptive mutations. Third, 36% of adaptive mutations were found to be synonymous mutations, confirming the increasingly recognized role of synonymous changes in adaptive evolution that may involve both translational and nontranslational mechanisms (Liu 2020; Agashe 2021; Bailey et al. 2021; Callens et al. 2021). All adaptive synonymous mutations except one converted frequently used codons in *R. solanacearum* into unusual ones (supplementary table S8, Supplementary Material online), which may explain their functional effects. Moreover, when inspecting gene annotations, we found that the 25 beneficial mutations target various biological functions (table 1). Strikingly, nearly 40% affect regulatory functions such as global transcription regulators (*efpR* and Rsc0965) (Capela et al. 2017), putative quorum quenching (RSp0595), signal transduction (RSc1598), unknown transcription regulator (RSc0243), protein dephosphorylation (Rsp1469), protein folding (*ppiB*), degradosome (*rhlE1*), and DNA methylation (RSc1982). Another significant proportion of mutations (28%) affects metabolism and transport genes (*phbB*, RSc0194, RSc2277, Rsp1116, RSp1417, RSp1422, and RSp1590), and three mutations are located in genes belonging to the type VI secretion system (T6SS) operon (*tssJ*, RSp0759, and Rsp0769). The observed genetic architecture (i.e., the large number of genes and molecular functions targeted by adaptive mutations) indicates that symbiotic traits are complex and constitute a large mutational target for adaptive evolution.

### Adaptive Mutations Predominantly Improve Nodulation Competitiveness

**Fig. 4.**
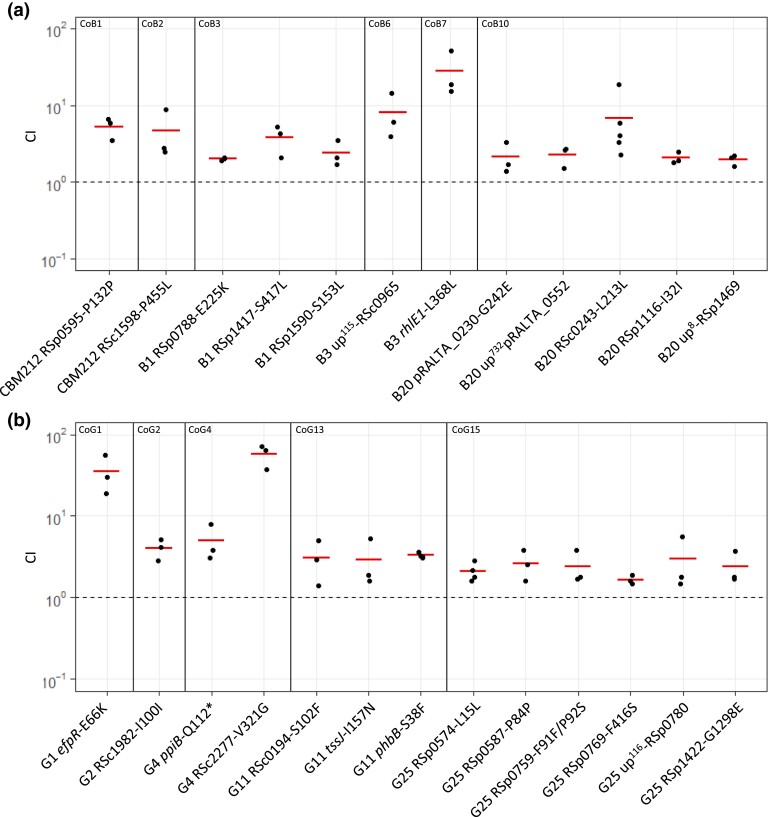
Nodulation competitiveness of adaptive mutants. Nodulation competitiveness effect of adaptive mutants from lineages B (*a*) and G (*b*). Evolved clones in which mutations were reconstructed are indicated at the bottom of the graph. Vertical lines separate the different mutational cohorts. Cohort numbers are indicated at the top of the graphs. CIs were calculated as the ratio of the number of nodules formed by the mutant strain on the number of nodules formed by the isogenic parental strain normalized by the frequency of each strain in the inoculum. Horizontal segments correspond to mean values of CI. Red segments indicate significantly beneficial mutations (*P* < 0.05, *t*-test with Benjamini–Hochberg correction). Data were obtained from three to four independent experiments. The sample size (*n*) is comprised between *n* = 3 and 4. Each CI value was obtained from 95 to 98 nodules harvested from 20 plants. The data underlying panels *a* and *b* and *P* values can be found in supplementary table S15, Supplementary Material online.

**Fig. 5.**
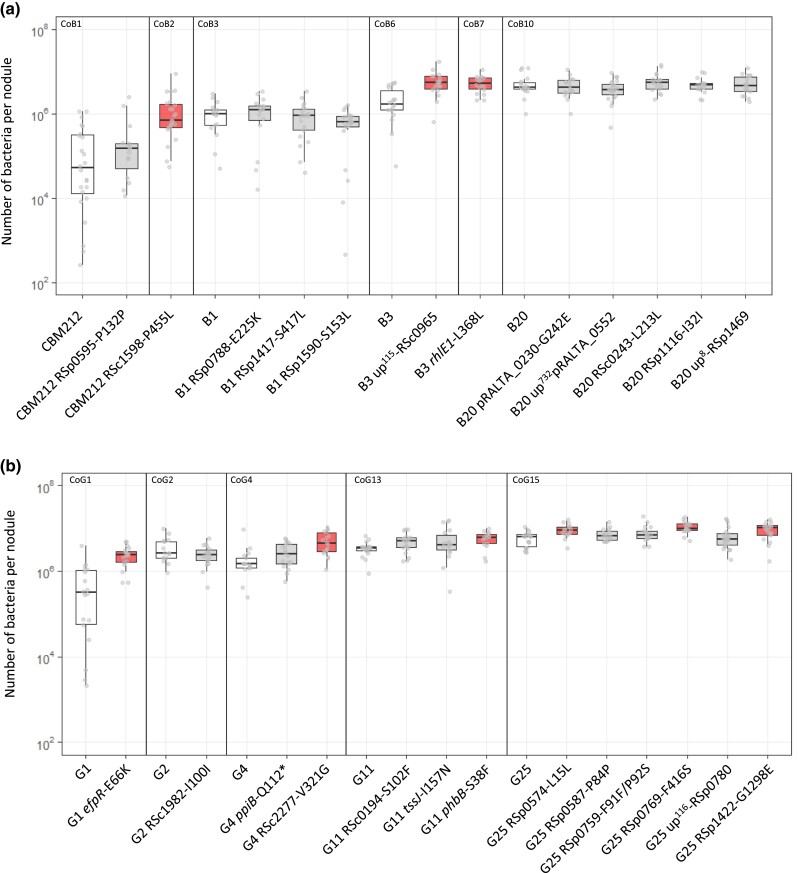
Within-host proliferation of adaptive mutants and corresponding isogenic parental clones. Distribution of the number of bacteria per nodule recovered for adaptive mutants from lineage B (*a*) and G (*b*) and their corresponding isogenic parental clones at 21 dpi. Rectangles span the first quartiles to the third quartiles, bold segments inside the rectangle show the median, unfiled circles represent outliers, and whiskers above and below each box show the largest and smallest values within 1.5 times interquartile range above and below the 75th and 25th percentile, respectively. Red boxes indicate the mutations that significantly improved within-host proliferation compared with the parental evolved clone (*P* < 0.05, Wilcoxon test with Benjamini–Hochberg correction) shown in the white boxes. Data were obtained from three to five independent experiments. For each experiment, nodules were harvested from six plants. The sample size (*n*) is comprised between *n* = 15 and 24. The data underlying panels *a* and *b* and *P* values can be found in supplementary table S16, Supplementary Material online.

Next, in order to uncover which symbiotic traits explain the observed fitness gain, we measured the effect of each adaptive mutation on the two traits contributing to the total symbiotic fitness, the nodulation competitiveness (i.e., the capacity of mutant bacteria to enter the root and induce nodule formation in competition with their corresponding parental strain) and within-host proliferation (i.e., the capacity of bacteria to multiply within each nodule). For reasons of experimental feasibility and to have sufficient data, the latter trait was measured in single inoculations and not in competitions with the parental strain. Since most (>90%) nodules accommodate a single strain (Daubech et al. 2017) and our nonnitrogen fixing strains are not supposed to be differentially affected by conditional sanctioning (Westhoek et al. 2021), we postulate that measures of within-nodule proliferation in single and coinoculation experiments should yield comparable outcomes. All mutations increasing symbiotic fitness improved nodulation competitiveness of evolved clones (fig. 4*a* and *b*). Gains in nodulation competitiveness ranged from 1.6-fold to 58-fold, with three mutations providing the highest gains: RSc2277-V321G (58-fold), *efpR*-E66K (35-fold), and *rhlE1*-L368L (28-fold). By contrast, within-host proliferation was improved by only a subset of adaptive mutations (fig. 5*a* and *b*). Only three mutations from lineage B (RSc1598-P455L, up^115^-RSc0965, and *rhlE1*-L368L) and six mutations from lineage G (*efpR*-E66K, RSc2277-V321G, *phbB*-S38F, RSp0574–L15L, RSp0769-F416S, and RSp1422-G1298E) significantly improved this fitness component. Moreover, gains in in planta proliferation were generally lower than gains in nodulation competitiveness and ranged from 1.6-fold to 6.4-fold. The highest proliferation gains were produced by the five mutations RSc1598-P455L (6.4-fold), *efpR*-E66K (3.7-fold), up^115^-RSc0965 (2.6-fold), RSc2277-V321G (2.5 fold), and *rhlE1*-L368L (2.4-fold), three of which also produced the highest nodulation gains.

To investigate whether improvements in nodulation competitiveness could be due to a better proliferation in the plant culture medium or rhizosphere, we measured the survival of the 25 mutants in competition with their isogenic parental strain in these two compartments. Results from these assays showed that none of the adaptive mutations improved bacterial ability to colonize the culture medium and only four of them, including up^115^-RSc0965, *efpR*-E66K, and *rhlE1*-L368L, improved the colonization of the rhizosphere (supplementary fig. S4, Supplementary Material online) although by smaller factors than improvements in nodulation competitiveness. These results showed that improvements in nodulation competitiveness were generally not associated with a better proliferation in the culture medium or of the rhizosphere, indicating that host entry is mainly controlled directly by the plant and is the dominant selective force driving the adaptation of legume symbionts in this evolution experiment.

### Evolutionary Modelling Predicts That Selection for Host Entry Drives Adaptation of Legume Symbionts

The preferential selection of nodulation competitiveness in our experiment prompted us to investigate the origin of this phenomenon. In particular, we wondered if stronger selection for host entry over within-host proliferation could be a general feature of symbiotic life cycles or, instead, if it is more likely to be a specific property of our experimental system that may arise due to genetic constraints on symbiotic traits and/or specific features of the selective regime. We used computer simulations to model the evolution of bacterial populations that cycle between two compartments: the external environment (rhizosphere) and the host tissues (root nodules). In our model, bacteria accumulate mutations in the rhizosphere due to transient hypermutagenesis (Remigi et al. 2014), although we consider that the bacterial population remains clonal within nodules (Remigi et al. 2014) (see supplementary file S1 and fig. S5, Supplementary Material online for additional details on the model). These assumptions are likely relevant not only to many rhizobium–legume interactions, given the high prevalence of error-prone DNA polymerases on rhizobial symbiotic plasmids (Remigi et al. 2014), but also to other horizontally transmitted symbioses where the hosts are exposed to highly diverse environmental bacterial populations and accommodate more homogenous populations within their tissues (Nyholm and McFall-Ngai 2004; Kumar et al. 2015; Chomicki et al. 2020; Fronk and Sachs 2022). In our experimental system, bacterial fitness is mediated by two phenotypic components: competitiveness to enter the host (resulting in nodulation) and within-host proliferation. Host entry imposes a very stringent selective bottleneck since 1) in most cases, each nodule is founded by one single bacterium (Gage 2002; Daubech et al. 2017), 2) there were usually between 100 and 300 nodules per lineage collected at each cycle (supplementary table S1, Supplementary Material online) while *∼*10^6^–10^7^ bacteria were inoculated at each cycle, and 3) bacterial genotypes differ in their competitive ability to form nodules (fig. 4*a* and *b*). Once inside the host, bacteria multiply to reach a carrying capacity that is directly proportional to their proliferation ability, before returning to the external environment.

**Fig. 6.**
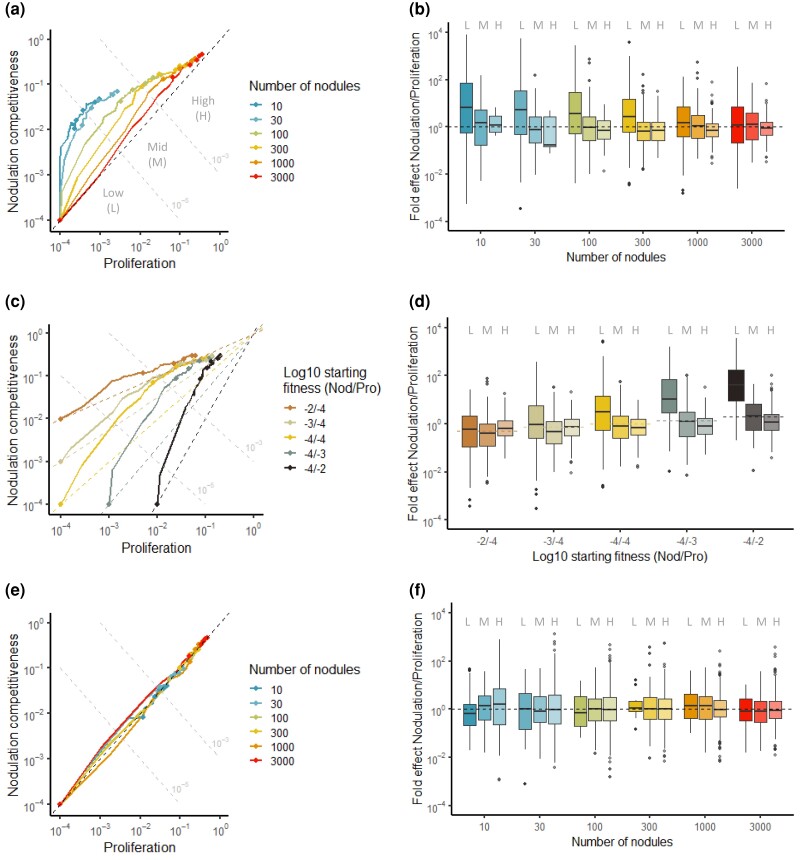
Relative strength of selection for nodulation competitiveness and within-host proliferation. (*a*, *c*, and *e*) Median fitness trajectories of 100 simulated populations evolving under different sizes of host entry bottleneck (10–3,000 nodules). Points indicate fitness values at cycles 0, 10, 20, 30, 40, and 50. Dotted grey lines represent iso-fitness lines (defined as the product of nodulation competitiveness and within-host proliferation values) used to delineate three fitness domains: low (L; fitness <10^−5^), mid (M; 10^−5^ <fitness <10^−3^), and high (H; fitness >10^−3^). The black dotted line represents the diagonal, along which population would improve both phenotypic traits equally well. (*b*, *d*, and *f*) Fold effect (nodulation competitiveness effect divided by proliferation effect) of mutations that reached a frequency of at least 30% in simulated populations. Color shading indicates the fitness domain (low, mid, or high) in which each group of mutations arose. Note that fold effects of mutations are indicated even when median fitness trajectories do not reach the high fitness domain (e.g., 10 and 30 nodule lines in *a*) because some individual replicate simulations (shown in supplementary figs. S8–S16, Supplementary Material online) do reach this fitness domain. The effect of different parameters on adaptive trajectories was tested: the size of host entry bottleneck (*a* and *b*), the initial fitness values of the ancestor (*c* and *d*), and the size of population bottleneck under a scenario where the bottleneck occurs after bacterial clonal proliferation (*e* and *f*). Raw data from evolutionary simulations are available at: https://doi.org/10.15454/QYB2S9.

When following the evolution of bacterial phenotypes in populations founded by an ancestor with low initial fitness (10^−4^ fold compared with the theoretical optimum, for each phenotypic component), we observed, in most cases, a faster increase in nodulation competitiveness relative to within-host proliferation (fig. 6*a*). Accordingly, the distribution of mutations that were selected at the early steps of the adaptive process was biased toward stronger improvement of nodulation competitiveness over proliferation (fig. 6*b*, low fitness domain). This dominance decreased and was sometimes reverted, as populations progressed toward high fitness values. Decreasing the strength of the host entry bottleneck also reduced the dominance of early selection for nodulation competitiveness, until abolishing it for the largest bottleneck size tested (3,000 nodules).

In our experiment, the ancestral nodulating strains had a much higher potential for improvement in nodulation competitiveness relative to proliferation (5 × 10^4^ vs. 10^2^–10^3^ fold, respectively, compared with the natural symbiont *C. taiwanensis*), which could explain the faster evolution of the former trait. We performed additional simulations to test how evolutionary trajectories are affected by the genotype of the ancestor. As expected, starting from a higher proliferation fitness in the ancestor further increased the overall fold effect of early selected mutations on nodulation competitiveness (fig. 6*c* and *d*). In contrast, starting with a higher initial nodulation competitiveness value of 10^−3^ or 10^−2^ in the ancestor was not sufficient to lead to a stronger selection for proliferation, further confirming the asymmetry between selective pressures acting on the two phenotypes. These findings were qualitatively robust to our assumptions regarding the genetic architecture of the two symbiotic phenotypes (supplementary file S1 and figs. S6–S15, Supplementary Material online).

Finally, we wondered if the chronology of symbiotic steps impacts on selective pressures and evolutionary trajectories. We modified the simulations so that clonal multiplication of bacteria (according to their proliferation fitness) occurs before the selective bottleneck. Although this model is not relevant in the context of the legume–rhizobium symbiosis, it may apply to other symbiotic or pathogenic life cycles, for example, when a selective bottleneck occurs during dissemination in the environment or within the host (Joseph et al. 2015; Hullahalli and Waldor 2021), as well as in the case of vector-mediated transmitted pathogens (Goodrich-Blair and Clarke 2007; Koinuma et al. 2020) or vertically transmitted symbionts (Perreau et al. 2021). In this context, the dominance of nodulation competitiveness was strongly reduced, bringing the two selective forces close to equilibrium (fig. 6*e* and *f* and supplementary fig. S16, Supplementary Material online), in agreement with the expectation that each fitness component should have a similar influence on adaptation when they have a multiplicative effect on global fitness. Therefore, the dominance of nodulation competitiveness in the previous simulation runs is likely a consequence of the selective bottleneck occurring before within-host bacterial multiplication and just after the transient hypermutagenesis phase in the rhizosphere.

Altogether, our simulations show that the selective pressures mediated by the plant that are experienced by symbiotic bacteria are asymmetric, with a dominance of selection for nodulation competitiveness over within-host proliferation. This asymmetry occurs when a genetically and phenotypically diverse bacterial population from the rhizosphere is exposed to the strong selective bottleneck at host entry, allowing the efficient selection of the most competitive clones by the host plant. The fact that the ancestor from our evolution experiment has a lower nodulation competitiveness value compared with proliferation (relative to that of the wild-type rhizobium *C. taiwanensis*) is an additional factor expected to have contributed to the stronger selection on nodulation competitiveness. Importantly, the key factors responsible for the dominance of selection for host entry competitiveness in our model (strong bottleneck at host entry and phenotypic diversity of environmental microbial populations) are found in other symbiotic systems, suggesting that competition for host entry is probably an important driver of bacterial adaptation in emerging symbiotic associations.

## Discussion

Horizontally transmitted symbiotic bacteria have complex lifecycles during which they have to face multiple environmental constraints, both outside and inside the host. Assessing the relative influence of each of these selective pressures on the adaptive trajectories of emerging symbiotic bacteria is critically needed to better understand the eco-evolutionary dynamics of microbial populations (Obeng et al. 2021). In previous work, Burghardt et al. showed, using multistrain rhizobium communities, that rhizobial fitness mainly depends on the selection imposed by plant hosts compared with the selection in the soil (Burghardt et al. 2018). Here, we analyzed the relative strength of selective pressures exerted by the plant on legume symbionts at different stages of the interaction. We show that selection on competitiveness for host entry is the dominant selective pressure shaping early adaptation of new rhizobia. This trait improved very fast during our evolution experiment, to a level comparable with that of the natural *Mimosa* symbiont *C. taiwanensis*, and all adaptive mutations identified in lineages B and G improved nodulation competitiveness. Evolutionary simulations led us to propose that the dominance of the selection on host entry over the selection on within-host proliferation that we observed in our system is strongly dependent on the strength of the selective bottleneck that occurs at host entry and its position in the life cycle of symbionts. These results shed light on the importance of selective bottlenecks in shaping the evolutionary trajectories of emerging legume symbionts. Most theoretical (Bergstrom et al. 1999; LeClair and Wahl 2018) and experimental (Sousa et al. 2017; Wein and Dagan 2019; Garoff et al. 2020; Mahrt et al. 2021; Windels et al. 2021; Izutsu et al. 2022) works on the influence of bottlenecks on microbial adaptation have focused on nonselective bottlenecks that randomly purge genetic diversity and reduce the efficiency of natural selection. Another aspect of bottlenecks emerges when considering that transmission and host colonization can be, at least partially, dependent on microbial genotype, prompting us to consider infection bottlenecks as selective events (Handel and Bennett 2008). Selective bottlenecks have already been described during virus infections (Wilker et al. 2013; Joseph et al. 2015; Moncla et al. 2016; Sobel Leonard et al. 2016; Huang et al. 2017) but received less attention in bacteria, except for their role in the evolution of phenotypic heterogeneity (Libby and Rainey 2011; Moxon and Kussell 2017; De Ste Croix et al. 2020; Schmutzer and Wagner 2020). Yet, potentially all host-associated bacterial populations (whether commensal, pathogenic, or mutualistic) experience bottlenecks of various intensities (sometimes going down to only a few cells, Gama et al. 2012) during their life cycles (Abel et al. 2015; Papkou et al. 2016; Fronk and Sachs 2022). Since competitiveness for host entry is a complex, polygenic phenotypic trait (Cossart and Sansonetti 2004; Bright and Bulgheresi 2010; Rowe et al. 2019; Wheatley et al. 2020; Visick et al. 2021) that often displays extensive variation within natural populations (Bongrand et al. 2020; Mendoza-Suárez et al. 2021), bottlenecks are expected to represent major selective events (instead of purely stochastic events) in many symbiotic life cycles. Interestingly, strong selection for host entry in experimentally evolved mutualistic and pathogenic symbionts was recently observed in two studies (Robinson et al. 2018; Bacigalupe et al. 2019) reinforcing the idea that selective bottlenecks at host entry may play a crucial role in the evolution of diverse host–microbe interactions. As a limitation of our study, we note that our experimental system differs from the life cycle of established rhizobia, where additional factors such as symbiotic nitrogen fixation or bacterial life in soil may represent additional significant selective pressures (Burghardt 2019; Batstone et al. 2020; Burghardt et al. 2022).

In multistep infection processes, the different infection steps can be functionally linked either by couplings or trade-offs (Ebert et al. 2016; Hall et al. 2019). Our study unveiled a new characteristic of the rhizobium–legume interactions: the recurrent genetic coupling between nodulation competitiveness and within-host proliferation. Indeed, all the nine identified mutations that improved within-host proliferation also improved nodulation competiveness, while the reverse was not true. This result corroborates our previous data showing that mutations promoting nodule cell infection always improved nodule formation (Marchetti et al. 2010; Guan et al. 2013). It is conceivable that the intracellular release and proliferation of rhizobia in nodule cells are dependent on the efficiency of the earliest symbiotic events, that is, the entry and progression of bacteria in ITs and the concomitant divisions of nodule cells preparing for the accommodation of bacteria. Consistently, delayed progression of ITs across the root cell layers was shown to impair the release of bacteria in nodule cells (Xiao et al. 2014). Moreover, the identification of plant receptors (LYK3, NFR1, Sym37, and SYMRK) and transcription factors (NIN, ERN1, and ERN2) involved in both nodule organogenesis and rhizobial intracellular infection (Capoen et al. 2005; Moling et al. 2014; Cerri et al. 2016; Liang et al. 2019; Liu et al. 2021) supports the existence of common mechanisms controlling the two processes on the plant side. Future functional analyses of the adaptive mutations identified in this study will expand our understanding of the molecular bases of nodulation competitiveness in rhizobia (Younginger and Friesen 2019; Bovin and Lepetit 2020; Mendoza-Suárez et al. 2021) and its relationship to within-host proliferation. Including mutualistic traits (nitrogen fixation and host growth promotion) in the analysis of the genetic couplings (or trade-offs) between the different symbiotic traits will be important to fully characterize the genetic constraints shaping the evolution of rhizobium–legume interactions (Younginger and Friesen 2019; Batstone et al. 2020; Quides et al. 2021). The genetic links between different phases of host colonization are generally poorly documented in other host–microbe associations, but they were analyzed in recent experimental evolution studies with opposite outcomes. Robinson et al. (2018) showed that early bacterial adaptation to zebrafish gut favored the improvement of host entry and interhost transmission without affecting within-host proliferation (Robinson et al. 2018). In another study, experimental evolution of *Vibrio fischeri* in symbiosis with squids led to the fixation of mutations in a global regulatory gene (*binK*) that improved both the initiation and maintenance of the interaction, through its action on several bacterial phenotypic traits (Pankey et al. 2017). Conversely, an example of trade-off between early and late infection stages was recently evidenced in *Xenorhabdus nematophila* interacting with insects (Cambon et al. 2019; Faucher et al. 2021). In the same line, a trade-off between within-vector and within-host fitness was evidenced in the mosquito-borne parasite *Plasmodium falciparum* causing malaria (Costa et al. 2018). These examples highlight the diversity and context dependence of genetic correlations that exist between bacterial phenotypic traits involved in symbiotic interactions.

In all lineages of this experiment, the rate of adaptation was very high during the first cycles of evolution and then tended to decrease over time. This classical pattern of evolution, due to diminishing return epistasis among beneficial mutations (Couce and Tenaillon 2015; Van den Bergh et al. 2018; McDonald 2019), was likely favored by the low initial fitness values of nodulating ancestors compared with the natural symbiont *C. taiwanensis* allowing the rapid acquisition of highly beneficial mutations during the first cycles (table 1). Elevated mutation rate in the rhizosphere, prior to the entry of bacteria in roots (Remigi et al. 2014), has also played an important role in the dynamic of adaptation by fueling the extensive genetic diversification of bacteria and exposing these populations to plant-mediated selection. A probable consequence of high mutation rate combined to strong selection is the presence of large cohorts of mutations that carry multiple beneficial mutations along with some neutral or slightly deleterious ones. Mutational cohorts were already described in previous evolution experiments (Lang et al. 2013; Maddamsetti et al. 2015), with cases of cohorts carrying two codriver mutations (Buskirk et al. 2017). Here, we identified up to six adaptive mutations in one of our cohorts. The occurrence of multidriver mutations per cohort might be explained by the nested, sequential fixation of multiple adaptive mutations in one lineage before it reaches detectable frequency (5% in our case) (Buskirk et al. 2017). This phenomenon, creating a “travelling wave” of adaptation (Neher 2013), was recently observed with high-resolution sequencing (Nguyen Ba et al. 2019) and is possibly amplified by the strong selection/high mutation regime of our experiment. Alternatively, it was proposed that, in the case of hypermutagenesis, the likelihood of multiple adaptive mutations arising simultaneously in a given genome becomes nonnegligible and might promote saltational evolution (Katsnelson et al. 2019).

In conclusion, our work shows that the relative strengths of the selective forces imposed by legumes strongly influence the evolutionary trajectory of symbiotic microbial populations. Selection for host entry has probably played a major role in the evolution of new rhizobia. This effect emerges from the presence of a selective bottleneck at host entry, although its importance can be modulated by other genetic and ecological factors. Investigating the role of selective bottlenecks in other host–microbe interactions will improve our ability to understand the evolutionary dynamics of microbial populations.

## Materials and Methods

### Bacterial Strains and Growth Conditions

Strains and plasmids used in this study are listed in the supplementary table S9, Supplementary Material online. *Cupriavidus taiwanensis* strains were grown at 28 °C on tryptone-yeast (TY) medium (Beringer 1974). *Ralstonia solanacearum* strains were grown at 28 °C either on rich medium (Boucher et al. 1985) containing bacto-peptone (10 g/L), yeast extract (1 g/L) and Casamino acids (1 g/L) or on minimal medium (Plener et al. 2010) supplemented with 2% glycerol. *Escherichia coli* strains were grown at 37 °C on lysogeny-broth medium (Bertani 1951). Antibiotics were used at the following concentrations: trimethoprim at 100 *µ*g/mL, spectinomycin at 40 *µ*g/mL, streptomycin at 200 *μ*g/mL, and kanamycin at 25 *μ*g/mL (for *E. coli*) or 50 *μ*g/mL (for *R. solanacearum*).

### Experimental Evolution

Symbiotically evolved clones and populations were generated as previously described (Marchetti et al. 2017). Five lineages, two (B and F) derived from the CBM212 ancestor, two (G and K) derived from the CBM349 ancestor, and one (M) derived from the CBM356 ancestor, previously evolved for 16 cycles using either 21-day cycles (B, G, and M lineages) or 42-day cycles (F and K lineages) of nodulation. We further evolved these lineages until cycle 35 using 21-day cycles of nodulation. For each lineage, at each cycle, 30 plants, grown in 15 Gibson tubes (two plants per tube) containing 20 mL of solid Fahraeus medium (Fahraeus 1957) and 40 mL of liquid Jensen medium (Jensen 1942), were inoculated with nodule bacterial populations from the previous cycle. Twenty-one days after inoculation, all nodules from the 30 plants were pooled, surface sterilized with 2.4% sodium hypochlorite for 15 min, rinsed with sterile water and crushed in 1 mL of sterile water. Then, 10% of the nodule crush resuspension was used to inoculate a new set of 30 plants the same day. Serial dilutions of each nodule crush were plated, and one clone was randomly selected from the highest dilution and purified. The selected clones, the rest of the nodule crushes, and a 24-h culture of bacteria from an aliquot of nodule crushes were stored at −80 °C at each cycle.

### Sequencing Bacterial Populations and Clones

Aliquots of frozen nodule bacterial populations or single colonies from purified clones were grown overnight in rich medium supplemented with trimethoprim. Bacterial DNA was extracted from 1 mL of culture using the Wizard genomic DNA purification kit (Promega). Evolved population DNAs were sequenced at the GeT-PlaGe core facility (https://get.genotoul.fr/), INRAE Toulouse. DNA-seq libraries have been prepared according to Illumina's protocols using the Illumina TruSeq Nano DNA HT Library Prep Kit. Briefly, DNA was fragmented by sonication, size selection was performed using SPB beads (kit beads), and adapters were ligated to be sequenced. Library quality was assessed using an Advanced Analytical Fragment Analyzer (Agilent), and libraries were quantified by quantitative PCR using the Kapa Library Quantification Kit (Roche). Sequencing has been performed on a NovaSeq6000 S4 lane (Illumina) using a paired-end read length of 2 × 150 bp with the Illumina NovaSeq Reagent Kits.

Evolved clones were resequenced either by C.E.A/IG/Genoscope using the Illumina GA2X technology (clones B8, B16, F16, G8, G16, K8, K16, M8, and M16) or by the GeT_PlaGe core facility using the Illumina technology HiSeq2000 (clones B1, B2, B3, B4, B5, B6, B7, B9, B10, B11, B12, B13, B14, B15, G1, G2, G3, G4, G5, G6, G7, G9, G10, G11, G12, G13, G14, G15, M4, and M12), HiSeq3000 (clones B20, B25, B30, G25, G30, K1, K2, K3, K4, K5, K6, K9, K10, K11, K12, K13, K14, K15, M2, M6, M10, and M14), MiSeq (K7), or NovaSeq6000 (clones B35, F35, G35, K35, and M35).

### Detection of Mutations and Molecular Analyses

Sequencing reads from NovaSeq6000 runs (all whole populations and clones from cycle 35) were mapped on the chimeric reference genome of the ancestral strain, comprising *R. solanacearum* GMI1000 chromosome (GenBank accession number: NC_003295.1) and megaplasmid (NC_003296.1) together with *C. taiwanensis* symbiotic plasmid pRalta (CU633751). Mutations were detected using breseq v0.33.1 (Deatherage and Barrick 2014) with default parameters, either using the polymorphism mode (for whole-population sequences) or the consensus mode (for individual clones). Mutation lists were curated manually in order to remove mutations present in the ancestral strains as well as false-positive hits arising from reads misalignments in low complexity regions. Moreover, alleles showing aberrant trajectories in the time-course whole-population sequencing data were checked manually, by inspecting either breseq output files and/or read alignments with IGV (Robinson et al. 2011), and corrected as needed. In this work, we focused our attention on SNPs and indels detected above 5% frequency in the populations. Recombinations, rearrangements, and insertion sequence movements were not analyzed exhaustively. Mutations detected in populations are listed in supplementary table S4, Supplementary Material online.

Sequencing data of the remaining clones were analyzed as described previously (Capela et al. 2017) with the PALOMA bioinformatics pipeline implemented in the MicroScope platform (Vallenet et al. 2020). The complete list of mutational events generated for these clones are available on the MicroScope platform (https://mage.genoscope.cns.fr/microscope/expdata/NGSProjectEvo.php, SYMPA tag). Mutations detected in clones are listed in supplementary table S10, Supplementary Material online.

In order to identify mutations forming temporal clusters (cohorts) through populations, we selected mutations having a frequency higher than 30% in at least one cycle and clustered their frequency using the *hclust* function in R v3.6.1 (R Core Team 2019). Then, we separated cohorts using a cutoff distance of 0.3.

Subpopulation genealogies in lineages B and G, shown as Muller plots in supplementary figure S3, Supplementary Material online, were reconstructed by comparing mutations found in cohorts and in individually sequenced clones. Cohorts for which ancestry cannot be ascertained (mutations not found in any clone) were not included in these plots. Relative frequencies of genotypes were calculated manually and plotted with the R package MullerPlot (Noble 2019).

G scores were calculated as described (Tenaillon et al. 2016). Briefly, for each protein-coding gene i, the expected number of mutations *E*_i_ = *N*_tot_ (*L*_i_/*L*_tot_) was calculated with *L*_i_ being the length of the protein-coding gene i, *L*_tot_ the sum of all protein-coding gene lengths, and *N*_tot_ the sum of mutations over all protein-coding genes. Since we found that synonymous mutations can be adaptive, we used all types of mutations in coding regions to calculate this statistics. Moreover, we included all mutations beyond 5% frequency in this analysis since we assumed that strong clonal interference may prevent adaptive mutations to rise to high frequency. Then, G scores were calculated as *G*_i_ = 2 *N*_i_log_e_ (*N*_i_/*E*_i_) with *N*_i_ corresponding to the number of independent mutations observed in that gene across all populations. To evaluate statistical significance of G scores, we ran 1,000 simulations where the total number of mutations used for the calculation of the observed G scores (3,330) were randomly assigned to the coding genes according to their respective length to compute simulated G scores for each bacterial gene (Tenaillon et al. 2016). The sum of simulated G scores was compared with the observed sum, and we computed a *Z* score and *P* value from these simulated G statistics. These simulations were also used to compute mean G scores for each gene and to calculate the associated *Z* scores and *P* values (adjusted using a Bonferroni correction).

### Reconstruction of Mutations in Evolved Clones

Mutant alleles and constitutively expressed reporter genes (GFPuv and mCherry) were simultaneously introduced into *Ralstonia* evolved clones by cotransformation using the MuGent technique (Dalia et al. 2014; Capela et al. 2017). Briefly, two DNA fragments were prepared, the first one from the pRCK-P*ps*-GFP or pRCK-P*ps*-mCherry linearized plasmids carrying an antibiotic (kanamycin) resistance gene allowing the integration of the resistance gene in the intergenic region downstream the *glmS* gene and the second one carrying the mutation to be introduced prepared by PCR amplification of a 6-kb surrounding region using genomic DNA of evolved clones as template and high fidelity Phusion polymerase (Thermo Fisher Scientific). Both were cotransformed into naturally competent cells of *Ralstonia* evolved clones grown in minimal medium for 48h at 28 °C. Cotransformants resistant to kanamycin were screened by PCR using primers specifically amplifying mutant or wild-type alleles and verified by Sanger sequencing. Primers used in mutation reconstructions are listed in supplementary table S9, Supplementary Material online.

### Analyses of Symbiotic Phenotypes on *M. pudica*

*Mimosa pudica* seeds (LIPME 2019 production obtained from one commercial seed [B&T World Seed, Paguignan, France] of Australian origin) were sterilized as described (Chen et al. 2003) by immersion in 95% H_2_SO_4_ for 15 min and 2.4% sodium hypochlorite solution during 10 min and rinsed in sterile distilled water five times. Seeds were soaked in sterile water at 28 °C under agitation for 24 h and then deposited on soft agar (9.375 g/L) and incubated at 28 °C during 24 more hours in darkness. Then, seedlings were cultivated in glass tubes (two seedlings per tube) under N-free conditions, each tube containing 20 mL of solid Fahraeus medium (Fahraeus 1957) and 40 mL of liquid Jensen medium (Jensen 1942) diluted 1:4 with sterile water. Plants were incubated in a culture chamber at 28 °C under a photoperiod day/night of 16 h/8 h and positioned randomly in the different experiments.

For symbiotic relative fitness and nodulation competitiveness assays, two strains of *R. solanacearum* expressing differential constitutive fluorophores (GFPuv or mCherry) or one strain of *R. solanacearum* and one strain of *C. taiwanensis*, both expressing differential antibiotic resistance (streptomycin and trimethoprim), were coinoculated onto *M. pudica* plantlets grown for 3–4 days in the culture chamber. Both strains were inoculated in equivalent proportion (*∼*5 × 10^5^ bacteria of each strain per plant) except for the comparisons of the *Ralstonia* nodulating ancestors with *C. taiwanensis* where strains were mixed in a *∼*500:1 ratio (*∼*10^4^ bacteria of *C. taiwanensis* and *∼*5 × 10^6^ bacteria of nodulating ancestors per plant). Nodules were harvested 21 days after inoculation, surface sterilized by immersion in 2.4% sodium hypochlorite solution for 15 min and rinsed with sterile water. For symbiotic relative fitness measurements, sterilized nodules from 20 plants (see the exact number of harvested nodules in supplementary file S1, Supplementary Material online) were pooled and crushed in 1 mL of sterile water and diluted and spread on selective solid medium using an easySpiral automatic plater (Interscience). After 2-day incubation at 28 °C, colonies were screened either by plating on selective medium in case of coinoculations of *Ralstonia* evolved clones with *C. taiwanensis* or based on fluorescence using a stereo zoom microscope (AxioZoom V16, Zeiss) for coinoculations of *Ralstonia* evolved clones with *Ralstonia* reconstructed mutants. For nodulation competitiveness assays, ∼96 individual nodules per experiment were crushed separately in 96-well microtiter plates, and droplets were deposited on selective medium. Nodule occupancy was determined by screening bacteria grown in the droplets either based on selective medium or based on their fluorescence as described for fitness measurements. Both nodulation competitiveness and relative fitness assays were measured in at least three independent biological replicates. CIs were calculated by dividing the ratio of the number of test strain (evolved strains or reconstructed mutants) versus reference strain (*C. taiwanensis* or evolved parental strains, respectively) in nodules normalized by the inoculum ratio. When CI values were all above 1, CI values were transformed by their inverse and compared with the value 1 using a one-sided Student *t*-test with Benjamini–Hochberg correction (*P* < 0.05).

For within-host proliferation assays, plantlets were inoculated with a single strain (5 × 10^5^ bacteria per plant). In each experiment, nodules from six individual plants were collected separately 21 days after inoculation, surface sterilized for 15 min in a 2.4% sodium hypochlorite solution, rinsed with sterile water, and crushed in 1 mL of sterile water. Dilutions of nodule crushes were plated on selective solid medium using an easySpiral automatic plater (Interscience). Two days after incubation at 28 °C, the number of colonies was counted. Within-host proliferation was estimated as the number of bacteria per nodule. For each strain, 15–24 measurements from three independent biological replicates were performed. Pairwise comparisons of proliferation values were compared using a two-sided Wilcoxon rank sum test with Benjamini–Hochberg correction.

For proliferation in Jensen culture medium and rhizosphere colonization assays, plantlets were coinoculated with pairs of strains expressing differential constitutive fluorophores (GFPuv or mCherry) in equivalent proportion (5 × 10^6^ bacteria of each strain per plant). Seven days after inoculation, bacteria present in the culture medium were diluted and plated on selective solid medium using an easySpiral automatic plater (Interscience). To isolate bacteria attached to the roots, roots were removed from the culture medium, placed in 4 mL of sterile water, and strongly vortexed for 1 min, diluting and plating on selective medium using an easySpiral automatic plater (Interscience). Two days after incubation at 28 °C, colonies were screened for fluorescence using a stereo zoom microscope (AxioZoom V16, Zeiss). CIs for proliferation in Jensen culture medium or rhizosphere colonization were calculated as described above. CI values were compared with the value 1 using a two-sided Wilcoxon rank sum test with Benjamini–Hochberg correction.

For dry weight measurements, aerial parts of plants were collected at 21 days after inoculation and dried for 2 days at 65 °C. Dry weights of individual plants are available in supplementary table S13, Supplementary material online.

Sample sizes are indicated in Supplementary material online containing raw data. We performed three or more independent biological replicates for each analysis. In cases where we performed only three independent replicates, each replicate was based on the analysis of a large number of plants or nodules. Independent biological replicates were performed on different days, with different plants and different bacterial cultures.

### Modelling/Simulations

Full details on evolutionary simulations and the list and values of parameters used in the simulations are available in supplementary file S1 and table S11, Supplementary Material online, respectively.

## Supplementary Material

msad116_Supplementary_Data

## Data Availability

Sequencing data are available under NCBI SRA BioProject ID PRJNA788708 and SRP353965. Raw experimental data are available as supplementary tables. Raw data generated from computer simulations are deposited on the Data INRAE dataverse (https://doi.org/10.15454/QYB2S9). Computer code used to analyze genomic data and to perform computer simulations are deposited on the Data INRAE dataverse (https://doi.org/10.15454/QYB2S9). ***Conflict of interest statement.*** The authors declare no competing interests.
