# Supplementary material for: A Selective Bottleneck During Host Entry Drives the Evolution of New Legume Symbionts
Source: Mol Biol Evol. 2023 May 15;40(5):msad116. doi: 10.1093/molbev/msad116 (PMC10220510; doi:10.1093/molbev/msad116)
Supplement: msad116_Supplementary_Data [file msad116_supplementary_data.zip › Supp_Info_EVOPOP_MBE.pdf]

Supplementary File 1: Model framework and supplementary simulations to assess the robustness to variations in the genetic architecture of symbiotic traits

May 22, 2023

1 Model framework

1.1 Ecology of symbiotic life cycles

We simulated bacterial populations that repeatedly cycle between two compartments, the rhizosphere and root nodules (within-host compartment; supplementary fig. S5). In the rhizosphere, bacteria undergo transient hypermutagenesis, a phenomenon increasing basal mutation rate by 10-100 times [1]. Although we previously estimated experimentally that bacteria undergo a few (< 5) divisions in the rhizosphere, we were unable to detect any sign of selection associated with these divisions. For simplicity, we thus assume in the simulations that bacteria do not reproduce in this compartment. Bacterial population size in the rhizosphere was set to $N_{rhizo} = 10^6$ cells. To induce and enter nodules, bacteria undergo a very stringent bottleneck. Indeed, we previously determined experimentally that 97% of *M. pudica* nodules contain only one single genotype when co-inoculated with 2 isogenic strains carrying different fluorescent reporters [2], and the number of nodules collected at each cycle of our evolution experiment was generally comprised between 100 and 225 (first and third quartile of the distribution of nodule numbers collected during the evolution experiment, supplementary table S1), since each plant forms multiple nodules and the experiment was run on pools of 20 or 30 plants. In the model, we thus considered that each nodule is founded by one single bacterium, and we studied the influence of the size of the nodulation bottleneck on adaptation by varying the total number of nodules formed by each population at each cycle (with nodule numbers ranging from 10 to 3000). Moreover, we also know from previous work that bacterial genotypes can significantly differ in their competitive ability to form nodules [3, 4]. Therefore, nodulation represents a selective bottleneck and we define nodulation competitiveness fitness value as the probability that a rhizospheric bacterium forms a nodule. Of note, the size of the nodulation bottleneck is a constant value set within each simulation run, and does not depend on nodulation competitiveness of the population, that evolves along successive cycles. In the model, each nodule is founded by a bacterial genotype sampled from the whole population, with a probability of each genotype forming a nodule equals to the product of its relative frequency in the population by its nodulation competitiveness fitness value. Once in the nodule, bacteria multiply clonally (mutation rate within nodules is negligible compared to that of the rhizosphere [1]). We previously showed that within host fitness values are also dependent on bacterial genotypes [4], so the number of each bacterial clone within nodule is set to their within-host proliferation fitness value multiplied by the maximum bacterial load per nodule, estimated to $N_{nod} = 10^8$ cells/nodule [2]). At the end of each cycle, bacteria are pooled from all the nodules within a given population and 10^6 cells are randomly sampled to re-inoculate the next cycle. This main version of the model is named "v1".

A variation from the main model was designed to test if the chronology of symbiotic events affects evolutionary dynamics. In this version of the model (named version "Inv1"), we simulate populations that multiply clonally (up to numbers defined by the within-host proliferation fitness value) before experiencing the nodulation bottleneck.

1.2 Evolution of bacterial phenotypes

Using the model from [5] as a base, we developed a multi-dimensional Fisher's Geometric Model to simulate the adaptive walk of bacteria. We decomposed bacterial fitness into two components (nodulation competitiveness and proliferation within nodules), each described by a phenotypic vector (x and y, respectively):

$$x = \{x_1, ..., x_k, z_1, ..., z_m\}$$

$$y = \{y_1, ..., y_l, z_1, ..., z_m\}$$

where k is the number of dimensions of nodulation-specific phenotypes, l is the number of dimensions of proliferation-specific phenotypes, and m is the number of dimensions of pleiotropic phenotypes (e.g. affecting both nodulation competitiveness and proliferation). These pleiotropic phenotypes can be used to simulate possible genetic couplings between the two components.

The fitness of a bacterial strain is calculated as $w = w_n \times w_p$ with

$$\begin{cases} w_n = & \exp(-\frac{1}{2}x^T\mathbf{V}_n^{-1}x) \\ w_p = & \exp(-\frac{1}{2}y^T\mathbf{V}_p^{-1}y) \end{cases}$$

the nodulation and proliferation fitness, respectively. Fitness is therefore maximal ($w = 1$) when all traits are zero. $\mathbf{V}_n$ and $\mathbf{V}_p$ are diagonal matrices whose diagonal elements, $\sigma^2_{n_i}$ and $\sigma^2_{p_i}$, determine how deviations from zero for a given trait impact w_n and w_p: the larger these elements, the least the trait influences fitness. For each simulation, the $\sigma^2_{n_i}$ ($\sigma^2_{p_i}$ respectively) elements are randomly drawn from an inverse gamma distribution inv-$\Gamma(\alpha_{fit}, \beta_{fit})$ which average and variance are given by $\beta_{fit}/(\alpha_{fit} - 1)$ and $\beta_{fit}/((\alpha_{fit} - 1)^2(\alpha_{fit} - 2))$, respectively.

Each single mutation will modify all phenotypic components (i.e. all values of vectors x and y) by random values drawn from $k + l + m$ independent centered Gaussian distributions. For each simulation, the variances of these Gaussian distributions are independently drawn from an inverse gamma distribution with parameters α_{mut} and β_{mut}.

1.3 Parameter values

The full list of parameter values used in the simulations is shown in S11 Table. Simulations were used to explore the effect of the size of the nodulation bottleneck and of the fitness of the ancestral strain on bacterial adaptation. We used nodulation bottlenecks of sizes B = 10, 30, 100, 300, 1000, or 3000 nodules. The combinations of ancestral fitness values of nodulation competitiveness and within-nodule proliferation ($w_n0\,/\,w_p0$) tested were (10^{-4}/ 10^{-4}), (10^{-3}/ 10^{-4}), (10^{-2}/ 10^{-4}), (10^{-4}/ 10^{-3}) and (10^{-4}/ 10^{-2}). We used ancestral fitness values of (10^{-4}/ 10^{-4}) when testing the effect of bottleneck size, and a bottleneck of 300 nodules when testing the effect of ancestral fitness values. The parameters that remained constant in all simulations were either set to match (or approach) experimental estimations (number of bacteria in the rhizosphere $N_{rhizo} = 10^6$, maximum number of bacteria per nodule $N_{nod} = 10^8$, mutation rate $\mu_{rhizo} = 10^{-2}$), or chosen arbitrarily so

that the adaptive walks proceed with a kinetics comparable to the ones observed experimentally ($\alpha_{fit} = 2.5,\ \beta_{fit} = 0.15,\ \alpha_{mut} = 2.04,\ \beta_{mut} = 0.0208$).

In addition, we tested if our hypotheses regarding the genetic architecture of symbiotic traits affected simulation results by modifying the distribution of fitness effects and the level of pleiotropy of simulated mutations. In Fisher's Geometric Model, the distribution of mutational effects is dependent on the number of dimensions of the phenotypic space [6] (parameters k, l, and m in our model, where k is the dimension of nodulation-specific phenotypes, l is the dimension of proliferation-specific phenotypes, and m is the dimension of pleiotropic phenotypes). While we used values of $k = l = 10$ and $m = 0$ in most simulations, the proportion of beneficial and deleterious mutations was altered by setting $k = l = 3$ or $k = l = 20$ (supplementary fig. S6). Partial pleiotropy between the 2 phenotypic components manifests itself by a positive correlation between mutational effects (supplementary fig. S7), which was achieved by using non-null values for m while maintaining a total number of dimensions constant (hence keeping the same proportion of beneficial or deleterious mutations). We used the following combinations of parameters: $k = l = 6$ and $m = 4$, and $k = l = 2$ and $m = 8$.

When analysing the 'Inv1' version of the model, we studied the effect of the size of the nodulation bottleneck (B = 10-3000); default values were used for all other parameters ($\omega_n 0 = 10^{-4}$, $\omega_p 0 = 10^{-4}$, $k = l = 10$ and $m = 0$).

1.4 Simulations

We performed 100 replicate simulations of 50 evolutionary cycles for each set of parameters tested. Each replicate simulation begins by defining the variance-covariance matrices of mutation and fitness effects and the exact fitness values of the ancestor. The latter was achieved by following two steps: (i) defining random vector values for phenotypic traits (from a normal distribution, $\mu = 0,\ \sigma = 0.02$) and (ii) finding a set of vector values that minimize the sum of squared differences to the target value of each fitness component ('optim' function in R). This procedure allowed us to start each simulation with randomized vectors of phenotypic values while still being able to set manually the values of both fitness components.

All information regarding population size and composition at each cycle was recorded during simulations. To generate figures, we computed the mean fitness values of each replicate population at each cycle, as well as the fitness effect of each mutation on the two phenotypic components. Mean population fitness values are shown either individually (supplementary figs. S8-S16) or as the median value of the 100 replicate simulations (fig. 6 of the main text; supplementary figs. S9, S10, S12-S15). To represent the relative fitness effect of selected mutations on the two phenotypic components, we computed the fold effect of each mutation on nodulation competitiveness over its effect on proliferation.

All simulations and data analyses were performed with R v3.6.1 [7] and the following packages: *mvtnorm* [8], *rlist* [9], *ggplot2* [10], *tydiverse* [11] and *stringr* [12]. R code to perform and analyse these simulations, together with simulations results needed to reproduce the figures, are available from the Data INRAE Dataverse : https://doi.org/10.15454/QYB2S9.

2 Supplementary simulations show that results are robust to variations in the genetic architecture of symbiotic traits

The main objective of these evolutionary simulations was to investigate the trajectory of bacterial adaptation to symbiotic life cycles, and its dependence on selected ecological and evolutionary pa-

rameters. In the main text of this manuscript, we focussed on the analysis of two parameters: the size of the nodulation bottleneck and the initial fitness values of the ancestor. Evolutionary simulations relied on a generic model of adaptation (Fisher's Geometric Model, FGM) that has proven to provide a good qualitative description of adaptation when compared to microbial evolution experiments [6]. However, this theoretical model makes assumptions on the genetic architecture of the phenotypic traits of interest (the distribution of fitness effects and the level of pleiotropy) that are very hard to measure experimentally, and we wondered if the choice of values for some of the specific evolutionary parameters used in this model could alter its predictions. Therefore, we wanted to assess the robustness of our results to these assumptions. We ran additional simulations to test the effect of (i) the number of phenotypic dimensions of the FGM (controlling the shape of the distributions of mutational effects, hence the proportion of beneficial, neutral, and deleterious mutations) and (ii) the level of pleiotropy of mutations (no pleiotropy vs. partial pleiotropy).

First, the number of dimensions for nodulation competitiveness and within-host proliferation was set to 3 or 20 (compared to 10 for fig. 6 in the main text). We observed that increasing the dimensions of phenotypic vectors from 3 to 20 decreased the proportion of beneficial mutations from ∼46% to ∼20%, respectively, in the low-fitness ancestor (supplementary fig. S6), and decreased the rate of adaptation (supplementary figs. S8-S13). We still observed an asymmetry between selection for nodulation competitiveness and within-host proliferation, that was most prevalent for smaller nodulation bottlenecks (supplementary figs. S8-S10) or when the initial nodulation competitiveness fitness was lower than the initial proliferation fitness (supplementary figs. S11-S13). These results support the conclusion that the asymmetry between selective forces in this experimental life cycle can be expected for phenotypic traits showing a wide diversity of genetic architectures. We note that a difference in the number of dimensions between nodulation competitiveness and proliferation within nodules could also induce such an asymmetry, since the trait with the lowest dimension would have access to more beneficial mutations and would likely improve faster. Although we cannot rule out that symbiotic traits have different distributions of fitness effects in our evolving lineages, simulation results indicate that a selective nodulation bottleneck (when occurring just after the genetic diversification phase) is a sufficient condition to promote the faster selection of nodulation competitiveness, and thus likely plays a role in explaining our experimental observations.

Another genetic factor that can affect evolutionary trajectories is the level of pleiotropy of mutations, *i.e.* the fact that one given mutation can alter the two traits. Pleiotropy can be qualified either as positive, when a mutation improves the two (or more) traits of interest, or as antagonistic, when a mutation improves one trait and worsens the other. Since our experimental results identified cases of positive pleiotropy (figs 4 and 5, main text), we ran additional simulations to test its effect on the evolutionary trajectories of bacterial populations. This was done by using non-zero values for the number of dimension of pleiotropy phenotypes ($m > 0$), which altered the distributions of fitness effects by introducing a positive correlation between the two phenotypes (supplementary fig. S7a-c). A moderate level of pleiotropy ($k = l = 6$ and $m = 4$) has little impact on adaptive trajectories (supplementary fig. S14). Increasing the level of pleiotropy ($k = l = 2$ and $m = 8$) decreases asymmetry between selection on the two phenotypic traits, and accelerates adaptation (supplementary fig. S15). This was expected since a higher correlation between the two traits leads to increased variance in the distribution of fitness effects (supplementary fig. S7d). Therefore, more mutations with high-fitness effects are available for natural selection to act on.

Conclusions and Discussion

FGM provides a simple framework that allows to reproduce some general patterns observed in experimental evolution studies. However, the estimation of evolutionary parameters from exper-

imental data is not straightforward, and it is thus difficult to generate quantitative predictions. Here we explored different combinations of evolutionary parameters to test if they affected the main results of our simulations. In particular, we modified the proportion of beneficial vs. deleterious mutations available for each phenotypic component, by changing the dimensionality of the model, and the degree of pleiotropy of individual mutations. Overall, these additional simulations qualitatively supported the conclusions presented in the main text, indicating their robustness to a range of assumptions regarding the genetic architectures of the two traits of interest.

Our simulation and experimental results indicate that selective bottlenecks can play a significant role in the evolution of symbiotic micro-organisms. While the literature on the evolution of pathogenicity acknowledges that dissemination can be an evolvable trait [13], the stochasticity associated with strong infection bottlenecks is seldom considered, although it can have important consequences [14]. Consider for example a bacterial population where two independent mutants arise, the first one having a 10-fold increase in competitiveness for host entry and the other one a 10-fold increase in within-host proliferation. In theory, these two mutants have the same fitness (product of competitiveness for host entry by within-host proliferation phenotypic values) and should have the same probability to invade the resident population. However, if the selective bottleneck is strong and occurs before proliferation within the host, the "competitiveness mutant" will have a much higher probability to avoid extinction (and thus, a higher "realized fitness") than the "proliferation mutant". On the contrary, when clonal expansion occurs before the bottleneck, these probabilities become equal, since the lower competitiveness of the "proliferation mutant" will be compensated for by the higher number of individuals. This process, explaining the asymmetry between selection for host entry and selection for within-host proliferation, is thus dependent on the chronology of events and will become prominent when adaptive mutants are rare and for strong bottleneck sizes (but disappears for weak bottlenecks due to averaging effects).

References

[1] Remigi P., D. Capela, C. Clerissi, L. Tasse, O. Bouchez, J. Batut, S. Cruveiller, E.P.C. Rocha and C. Masson-Boivin. (2014) Transient hypermutagenesis accelerates the evolution of legume endosymbionts following horizontal gene transfer. PLOS Biology 12(9):e1001942.

[2] Daubech B., P. Remigi, G. Doin de Moura, M. Marchetti, C. Pouzet, M.C. Auriac, C.S. Gokhale, C. Masson-Boivin and D. Capela. (2017) Spatio-temporal control of mutualism in legumes helps spreading symbiotic nitrogen fixation. Elife 12(6):e28683

[3] Guan S., C. Gris, S. Cruveiller, C. Pouzet, L. Tasse, A. Leru, A. Maillard, C. Medigue, J. Batut, C. Masson-Boivin and D. Capela. (2013) Experimental evolution of nodule intracellular infection in legume symbionts. ISME J. 7(7):1367-77

[4] Doin de Moura GG., P. Remigi, C. Masson-Boivin, and D. Capela. (2020) Experimental Evolution of Legume Symbionts: What Have We Learnt? Genes 11(3):339

[5] Tenaillon O., B. Toupance, H. Le Nagard, F. Taddei and B. Godelle (1999) Mutators, Population Size, Adaptive Landscape and the Adaptation of Asexual Populations of Bacteria Genetics. 152:485-493

[6] Tenaillon O. (2014) The Utility of Fisher's Geometric Model in Evolutionary Genetics. Annu. Rev. Ecol. Evol. Syst. 45:179-201

[7] R Core Team (2019). R: A language and environment for statistical computing. R Foundation for Statistical Computing, Vienna, Austria. URL https://www.R-project.org/.

[8] Genz A., F. Bretz, T. Miwa, X. Mi, F. Leisch, F. Scheipl and T. Hothorn (2020). mvtnorm: Multivariate Normal and t Distributions. R package version 1.1-1. URL http://CRAN.R-project.org/package=mvtnorm

[9] Ren K. (2016). rlist: A Toolbox for Non-Tabular Data Manipulation. R package version 0.4.6.1. https://CRAN.R-project.org/package=rlist

[10] Wickham H. (2016) ggplot2: Elegant Graphics for Data Analysis. Springer-Verlag New York. https://ggplot2.tidyverse.org

[11] Wickham H. et al., (2019). Welcome to the tidyverse. Journal of Open Source Software, 4(43), 1686. https://doi.org/10.21105/joss.01686

[12] Wickham H. (2019). stringr: Simple, Consistent Wrappers for Common String Operations. R package version 1.4.0. https://CRAN.R-project.org/package=stringr

[13] Alizon S. and Y. Michalakis (2015) Adaptive virulence evolution: the good old fitness-based approach. Trends Ecol. Evol. 30:248-254.

[14] Papkou A., C. Gokhale, A. Traulsen and H. Schulenburg (2016) Host parasite coevolution: why changing population size matters. Zoology. 117:330-338.
